# Can Machine Learning with IMUs Be Used to Detect Different Throws and Estimate Ball Velocity in Team Handball?

**DOI:** 10.3390/s21072288

**Published:** 2021-03-25

**Authors:** Roland van den Tillaar, Shruti Bhandurge, Tom Stewart

**Affiliations:** 1 Department of Sports Sciences, Nord University, 7600 Levanger, Norway; 2 Sports Performance Research Institute New Zealand, Auckland University of Technology, Auckland 1010, New Zealand; shruti.bhandurge@aut.ac.nz (S.B.); tom.stewart@aut.ac.nz (T.S.); 3 Human Potential Centre, Auckland University of Technology, Auckland 1010, New Zealand

**Keywords:** handball, throwing velocity, artificial intelligence, inertial sensors

## Abstract

Injuries in handball are common due to the repetitive demands of overhead throws at high velocities. Monitoring workload is crucial for understanding these demands and improving injury-prevention strategies. However, in handball, it is challenging to monitor throwing workload due to the difficulty of counting the number, intensity, and type of throws during training and competition. The aim of this study was to investigate if an inertial measurement unit (IMU) and machine learning (ML) techniques could be used to detect different types of team handball throws and predict ball velocity. Seventeen players performed several throws with different wind-up (circular and whip-like) and approach types (standing, running, and jumping) while wearing an IMU on their wrist. Ball velocity was measured using a radar gun. ML models predicted peak ball velocity with an error of 1.10 m/s and classified approach type and throw type with 80–87% accuracy. Using IMUs and ML models may offer a practical and automated method for quantifying throw counts and classifying the throw and approach types adopted by handball players.

## 1. Introduction

Handball is a popular sport with around 20 million competitors worldwide. The physical attributes of the sport include a high playing tempo, a high volume of throws, frequent physical contacts, collisions, and rapid changes of movement [1,2]. To excel in handball, athletes need to train frequently, often daily for elite players. Extensive training load is detrimental to player performance and heightens injury risk. This can cause cumulative tissue overload [3], which is a contributing factor to overuse injuries. Shoulder injuries are highly prevalent in handball players [4]. Myklebust et al. [5] reported that 44–75% of the athletes he studied had a history of shoulder pain and 28% reported shoulder pain weekly [4]. In baseball (another overhead sport with throwing that is somewhat comparable to handball), half of youth pitchers reported shoulder pain during the competition season [6,7]. It is also reported that shoulder pain has an impact on athletes’ training activities [4,5,8], performance, and daily life [5].

One of the major risk factors for elbow and shoulder injuries is training load (undertraining and overtraining). Although it is hypothesized that restricting workloads may minimize the likelihood of athlete injury, reducing workloads in competition and training may also be detrimental to an athlete’s conditioning and performance [9]. Several studies have tried to quantify workload during training and competition to monitor this under- and overtraining [10,11]. Global positioning systems (GPS) have commonly been used to measure workload [12,13], but GPS can only measure whole-body velocity and acceleration in an outdoor environment and cannot measure throwing-related loads.

Specific to handball, there are usually six playing positions (left wing, left back, center back, right back, right wing, and pivot) during offensive or defensive phases [14]. The specificity of each position means there are different types of throws and passes, which adds complexity to workload monitoring. Besides the number and type of throws, the velocity of these throws is indicative of intensity and is, therefore, an important component of workload monitoring. Given the constant need to monitor external workload, there is a need to bridge the gap between time-intensive traditional methods and workload monitoring.

IMUs are increasingly used as a reliable and accurate method of monitoring external workloads [15,16]. They are light, inexpensive, and not restricted to laboratory spaces or indoor environments. Hence, they are easy to use during competitions across different sports [17,18]. In recent years, these microtechnology units have been used with machine learning methods to identify the number of throws in baseball [19] and deliveries in cricket [20]. In handball, only two studies have used IMUs to estimate throwing velocity [21,22]. Both studies showed that it was possible to estimate velocity with an accuracy of 1.3–2 m/s. However, neither study investigated if it was possible to identify different types of throws. In a preliminary study, an attempt was made to identify different types of throws with limited machine learning models [23]. However, only IMUs with a 16 g measurement range were utilized, and not IMUs with a higher range such as 200 g (which are more expensive compared to 16 g). A higher threshold may be more sensitive in detecting different throws and estimating ball velocity [16].

Machine learning techniques could potentially be used to monitor the number, types, and velocity of throws (intensity of the throws) automatically using IMU data. Therefore, the aim of this study was twofold: (1) to determine the accuracy of ML models for detecting throw type and ball velocity from IMU data and (2) to determine if accuracy increases when using a 200 g IMU compared to a 16 g IMU.

## 2. Materials and Methods

### 2.1. Participants

For this study, seventeen experienced handball players were recruited (10 men and 7 women, age 28.0 ± 7.3 years, body mass 74.4 ± 13.6 kg, body height 1.77 ± 0.09 m, handball training experience 17.7 ± 9.6 years). Data were collected across two testing sessions. The study complied with the current ethical regulations for research, conformed to the latest revision of the Declaration of Helsinki, and was approved by the Norwegian Centre for Research Data.

### 2.2. Procedure

After a standard warm-up, all participants threw with either a circular or whip-like wind-up from a standing, running, and jumping set-up [24]. These throws were made from 7 m in front of a standard handball goal (2 × 3 m). A total of 7–10 throws were performed for each combination (example: standing whip-like and running whip-like), which were randomized. Thus, 49–60 throws in total were performed by each participant.

Attached to the distal dorsal side of the throwing arm (IMeasureU, Auckland, New Zealand) was a wireless 9 degrees of freedom IMU containing a 3-axis accelerometer (low-g: ±16 g range, 1125 Hz sampling frequency; high-g: ±200 g, 1600 Hz), integrated with a magnetometer (range ±4900 µT, 100 Hz) and a 3-axis gyroscope (±2000 °/s, 1125 Hz). A Doppler radar gun (Stalker ATS II, Applied Concepts Inc., Plano, TX), located 11 m away from the handball goal—with a straight line between the target, the thrower, and the gun—was used to measure peak ball velocity. Speed was measured with an accuracy of 0.028 m/s within a 10° field. All testing sessions were also recorded by two video cameras: one from behind the goal and one from the side for confirming the type of throws.

All data processing and analysis were performed in R (version 4.0.2). Using linear interpolation, the low-g accelerometer and gyroscope data were resampled to 1150 Hz, and the high-g accelerometer data were resampled to 1600 Hz, given the sampling frequency fluctuation among sensors. Throwing events were then recognized using a peak detecting algorithm with a 1500°/s threshold on the gyroscope *y*-axis. Acceleration and rotational properties of each throw were calculated within different window sizes (2 s, 3 s, 4 s, 6 s) centered on the peak rotation event. The features calculated within each window included axis means, variance, power, amplitude, autocorrelation, skewness, kurtosis, coefficient of variation, and between-axis correlations. These were calculated for each axis (x, y, z) and the vector magnitude √(x^2^ + y^2^ + z^2^), for the high-g and low-g data separately. In total, 201 signal features were computed for each throw, within each of the four window sizes.

### 2.3. Modelling and Analysis

The machine learning modelling occurred in two parts. First, four different supervised machine learning models—the random forest (RF), linear support vector machine (SVML), support vector machine with a polynomial kernel (SVM-P), and gradient boosting machine (GBM)—were used to classify throw type (circular or whip-like) and throw approach type (running, standing, or jumping) from the signal features. Second, the same four models were used to estimate ball velocity (m/s), as measured by the radar gun. These four modelling algorithms were selected as they can be used for both classification (throw and approach type) and regression (ball velocity) problems. To evaluate the advantage of the high-g measurement range, these models were fit with the high-g and low-g feature sets separately.

Prior to modelling, highly correlated features were removed from the feature set if their correlation coefficient was above 0.95 [25]. All features were then standardized by subtracting their mean and dividing by their standard deviation. The optimal hyperparameters for each model were identified via 10-fold cross-validation using the *caret* R package [26]. Given the sample size of our study, the predictive accuracy of the final model (with the optimal hyperparameters) was estimated using leave-one-subject-out cross-validation. Feature importance of the optimal models was estimated using the permutation method (comparing model performance with and without each feature) using the *mlr* R package [27]. The importance was scaled to 0–100, so the importance of each feature was relative to the most important feature. For the classification models (i.e., throw and approach type) the sensitivity, specificity, balanced accuracy (mean of sensitivity and specificity), and F1 score were calculated for each predicted category. For the regression models (i.e., ball velocity), the root-mean-square error (RMSE), mean absolute error (MAE), and mean absolute percent error (MAPE) metrics were computed.

The results from each iteration of the leave-one-subject-out cross-validation were used within a two-way repeated-measures ANOVA to compare the F1 score (for approach type and throw type, separately) and mean absolute error (for ball velocity) across the four models and the high-g and low-g measurement ranges. Model assumptions (i.e., no significant outliers, dependent variable normality, sphericity) were checked prior to fitting each model using the *afex* and *performance* R packages. All three models violated the sphericity assumption and were thus adjusted using the Greenhouse–Geisser correction. Model-estimated means and contrasts between high-g and low-g measurement ranges were estimated using the *emmeans* R package, with multiple comparisons adjusted using the Holm method. A priori alpha of 0.05 was used for all analyses.

## 3. Results

The main peak ball velocities measured with the radar gun were as follows: standing: 20.7 ± 2.6 (circle), 20.1 ± 2.5 (whip); running: 22.0 ± 2.4 (circle), 21.2 ± 2.4 (whip); jumping: 21.4 ± 2.5 (circle), 21.0 ± 2.4 (whip). The GBM with a 3 s window was the most accurate model for classifying both approach type and throw type. The GBM model with the high-g features performed slightly better for classifying throw type, although this was not a significant difference (+2.4% balanced accuracy, *p* = 0.092). For approach type, the model with the low-g features performed better for classifying running (+6.6% balanced accuracy, *p* = 0.029), but not standing (+3.9%, *p* = 0.114) or walking (+3.8%, *p* = 0.114). The sensitivity, specificity, balanced accuracy, and F1 score for each approach and throw type are shown in Table 1.

Table 2 presents the ball velocity prediction error for each model tested. The best model for predicting ball velocity was the SVM-P with a 2 s window (mean absolute error = 1.1 m/s) using the high-g measurement range. For the 2 s window, models using the high-g measurement range features performed better at predicting ball velocity compared to the models with low-g features (RF, estimated mean absolute error difference = −0.15, *p* = 0.07; SVM-P, −0.22, *p* = 0.013; SVM-L, −0.14, *p* = 0.096; GBM, −0.21, *p* = 0.018).

**Figure 1:**
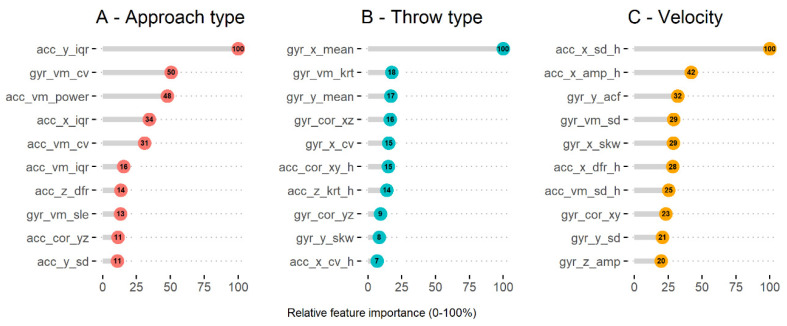
Relative signal feature importance for predicting approach type, throw type, and ball velocity. Models are as follows: (**A**) GBM with 3 s window and low-g range; (**B**) GBM with 3 s window and high-g range; (**C**) support vector machine with a polynomial kernel (SVM-P) with 2 s window and high-g range. The prefix indicates the accelerometer (acc) or gyroscope (gyr) sensor, the middle character is the axis (x, y, z, or vector magnitude), and the suffix indicates the feature: cv = coefficient of variation, skw = skewness, krt = kurtosis, dfr = dominant frequency, amp = peak-to-peak amplitude, ent = entropy. Features ending in “h” are obtained from the high-g accelerometer.

The most important signal feature for predicting approach type was the interquartile range of the accelerometer *y*-axis, while the mean and sum of the gyroscope *x*-axis (movement in sagittal plan) were important for throw type (Figure 1). The most important signal features for predicting ball velocity were the standard deviation of the accelerometer *x*-axis, and the amplitude of the gyroscope *x*-axis (Figure 1).

## 4. Discussion

This study investigated whether an IMU and machine learning could detect different types of team handball throws and predict ball velocity. Throwing was measured using an IMU and a radar gun during standing, running, and jumping throws with a circular and whip-like wind-up. The main findings were that the IMU data coupled with machine learning algorithms could predict peak ball velocity with an error of 1.1 m/s and that approach types and throw types could be classified with ~79–87% accuracy. Furthermore, signal features computed from the high-g data were better at predicting ball velocity compared to the models with low-g features.

The SVM-P was the best model to predict ball velocity when using a 2 s window (MAE of 1.10 m/s). This prediction was more accurate than in the two previous studies on throwing velocity prediction in handball [21,22], which was likely due to the higher IMU sampling rate in the present study (1600 vs. 500 Hz). Furthermore, it is also likely the low measurement range (±16 g) of the accelerometer affected the ball velocity prediction accuracy, as we showed a higher resolution sensor (±200 g) resulted in higher accuracy (1.1 vs. ~1.32 m/s). The error of the peak ball speed between the radar gun and the IMU measurements was 1.1 m/s, which could be less when the throws are directed straight towards the goal (3 × 2 m). Some throws were aimed at the corner or outside the goal and thereby resulted in an angle larger than 10 degrees from the midline of the radar gun. This probably resulted in lower peak velocities since the radar gun only measures the horizontal displacement to the goal. In future studies, a smaller target (0.5 × 0.5 m) could be used to gather more accurate radar gun readings [28].

To the best of our knowledge, this is the first study in handball that attempts to identify different types of throws, which makes it difficult to compare our findings with previous studies. The GBM model with a 3 s window was the most accurate model for classifying both approach type and throw type. The classification accuracy of the different throws was lower compared with a comparable study (93–97.4%) in tennis [18]. This could be due to the number of throws that were tested (962 throws vs. 28,582 shots), which makes it possible to develop a more robust model. Furthermore, the athletes in the present study were not elite players and did not have a lot of experience in the whip-like wind-up throwing technique. Due to this inexperience, some throws of different types looked very similar. Therefore, elite-level players who can perform all throwing techniques accurately should be included in future work.

The main limitation of the present study was that the handball throws were conducted in a standardized situation without any opposition or time limitations. In handball training and competition, the different throws are almost never standard, except with a penalty throw. Furthermore, handball throws can be divided into shots to the goal and passes to each other. These differences were not considered in the current study and should be considered in future studies. Therefore, the methods used in this study should be trialed in training and competition settings. This has been done in cricket training [20]. Furthermore, it should be investigated if lower sampling frequencies (e.g., 50 Hz) [29] can be used without compromising model performance, as IMUs with a lower sampling frequency can be a more cost-effective option.

In summary, using IMUs and ML models in a standardized situation may offer a practical and automated method for quantifying throw counts and classifying the throw and approach types adopted by handball players. This may enable players and coaches to monitor intensity and types of throws (and therefore workload) to potentially help to prevent injuries and improve performance.

## Figures and Tables

**Table 1:** Approach type classification accuracy for high-g and low-g measurement range.

Range	Type	Class	Sensitivity	Specificity	Balanced Accuracy	F1
High-g	Approach	Jumping	0.76	0.94	0.85	0.80
		Running	0.78	0.81	0.79	0.75
		Standing	0.78	0.86	0.82	0.79
	Throw	Circle	0.89	0.75	0.82	0.86
		Whip	0.75	0.89	0.82	0.79
Low-g	Approach	Jumping	0.79	0.95	0.87	0.82
		Running	0.83	0.85	0.84	0.81
		Standing	0.81	0.88	0.84	0.81
	Throw	Circle	0.84	0.75	0.80	0.84
		Whip	0.75	0.84	0.80	0.76

Note: Results from the optimal model, gradient boosting machine (GBM) with 3 s window.

**Table 2:** Ball velocity prediction error for each model, window size, and measurement range.

		High-g			Low-g		
Window	Model	MAE	MAPE	RMSE	MAE	MAPE	RMSE
2 s	RF	1.28	6.01	1.64	1.36	6.37	1.72
	SVM-P	1.10	5.13	1.45	1.31	6.06	1.75
	SVM-L	1.23	5.77	1.64	1.41	6.63	1.78
	GBM	1.23	5.78	1.59	1.40	6.48	1.84
3 s	RF	1.26	5.88	1.60	1.38	6.44	1.73
	SVM-P	1.18	5.55	1.52	1.39	6.56	1.74
	SVM-L	1.27	6.00	1.67	1.36	6.41	1.71
	GBM	1.28	5.96	1.69	1.52	7.04	1.96
4 s	RF	1.23	5.76	1.58	1.39	6.48	1.75
	SVM-P	1.20	5.61	1.54	1.32	6.21	1.68
	SVM-L	1.30	6.11	1.80	1.31	6.16	1.67
	GBM	1.29	6.04	1.66	1.42	6.62	1.80
6 s	RF	1.26	5.90	1.60	1.39	6.49	1.75
	SVM-P	1.28	6.01	1.68	1.36	6.29	1.86
	SVM-L	1.46	6.82	2.03	1.36	6.41	1.75
	GBM	1.32	6.16	1.70	1.54	7.16	1.95

## Data Availability

The data presented in this study are available on request from the corresponding author. The data are not publicly available due to national laws of the Norwegian government on privacy.
